# Colored Texture Analysis Fuzzy Entropy Methods with a Dermoscopic Application

**DOI:** 10.3390/e24060831

**Published:** 2022-06-15

**Authors:** Mirvana Hilal, Andreia S. Gaudêncio, Pedro G. Vaz, João Cardoso, Anne Humeau-Heurtier

**Affiliations:** 1 Univ Angers, LARIS, SFR MATHSTIC, F-49000 Angers, France; andreia.gaudencio@student.uc.pt (A.S.G.); anne.humeau@univ-angers.fr (A.H.-H.); 2 LIBPhys, Department of Physics, University of Coimbra, P-3004-516 Coimbra, Portugal; pvaz@uc.pt (P.G.V.); jmrcardoso@uc.pt (J.C.)

**Keywords:** colored texture analysis, dermoscopy, entropy, fuzzy entropy, information theory, medical image analysis, melanoma, texture analysis

## Abstract

Texture analysis is a subject of intensive focus in research due to its significant role in the field of image processing. However, few studies focus on colored texture analysis and even fewer use information theory concepts. Entropy measures have been proven competent for gray scale images. However, to the best of our knowledge, there are no well-established entropy methods that deal with colored images yet. Therefore, we propose the recent colored bidimensional fuzzy entropy measure, $FuzEnC_{2D}$, and introduce its new multi-channel approaches, $FuzEnV_{2D}$ and $FuzEnM_{2D}$, for the analysis of colored images. We investigate their sensitivity to parameters and ability to identify images with different irregularity degrees, and therefore different textures. Moreover, we study their behavior with colored Brodatz images in different color spaces. After verifying the results with test images, we employ the three methods for analyzing dermoscopic images of malignant melanoma and benign melanocytic nevi. $FuzEnC_{2D}$, $FuzEnV_{2D}$, and $FuzEnM_{2D}$ illustrate a good differentiation ability between the two—similar in appearance—pigmented skin lesions. The results outperform those of a well-known texture analysis measure. Our work provides the first entropy measure studying colored images using both single and multi-channel approaches.

## 1. Introduction

Texture features are of the utmost importance in segmentation, classification, and synthesis of images, to cite only few image processing steps. However, no precise definition of texture has been adopted yet. Texture is often referred to as the visual patterns appearing in the image. Several algorithms have been proposed for texture feature extraction in recent years and this research area is still the subject of many investigations [1,2,3,4,5,6,7,8,9,10]. Recently, seven classes were proposed to classify the texture feature extraction methods [1]: statistical approaches (among which we can find the co-occurrence matrices), structural approaches, transform-based approaches (Fourier transform-based approaches, among others), model-based approaches (such as the random field models), graph-based approaches (such as the local graph structures), learning-based approaches, and entropy-based approaches. The latter two classes (learning-based approaches and entropy-based approaches) are the most recent ones. Several studies have shown that the entropy-based measures are promising for texture analysis [11,12,13,14,15,16,17,18]. However, these studies are only at their beginning. Even if they have the great advantage of relying on reliable unidimensional, 1D, entropy-based measures (issued from the information theory field), they have the drawback—for most of them—of being designed for gray scale images only.

Besides texture, color is essential not only for human perception of images but also for digital image processing [19,20,21,22,23,24,25]. Unlike the intensity that is translated as scalar gray values for a gray scale image, color is a vectorial feature that is appointed to each pixel for a colored image [19]. In contrast to gray scale images that could be handled in a straightforward manner, colored images could be analyzed in several possible ways. This depends on many factors, such as the need to analyze texture or color, separately or combined, directly from the image or through a transformation, among other factors [19,24,25,26]. Only a few studies have been performed on colored texture analysis and most of them were achieved by adapting the application of gray scale textures analysis methods [13,18,27,28]. Nevertheless, color and texture are probably the most important components of visual features. Many biomedical images are color-textured: dermoscopy images, histological images, endoscopy data, fundus and retinal images, among others.

According to the World Health Organization, one in every three diagnosed cancer cases is a skin cancer and the incidence rate has been increasing over recent years. A non-invasive imaging modality, dermoscopy or epiluminescence microscopy (ELM), is one of the well-known non-invasive techniques used for skin cancer diagnosis on which most research studies are conducted. However, visual diagnosis alone might be misleading and subjective even when performed by experts. Thus, dermoscopy image analysis (DIA) using computer-aided diagnosis (CAD) systems is essential to help medical doctors. Several studies proposed computer extracted texture features for cutaneous lesions diagnosis, specifically for the most aggressive type, melanoma [29,30,31]. Melanoma is metastatic, thus its early diagnosis and excision would definitely increase the survival rate. Some DIA methods focus only on the dermoscopic image structure/patterns [32,33], others rely on colors [34,35,36], and some consider both [37], for more details please refer to [29,30,31]. Nevertheless, most studies propose learning-based approaches and only few have suggested entropy-based measures until now.

In this paper, we, therefore, propose novel bidimensional entropy-based measures dedicated to color images in their two approaches: single-channel approach, $FuzEnC_{2D}$, and multi-channel approaches, $FuzEnV_{2D}$ and $FuzEnM_{2D}$. First, we test the abilities of our proposed measures in colored texture analysis on different kinds of image. After that, we illustrate their application in the biomedical field by processing dermoscopic images of two different kinds of common pigmented lesions: melanoma and benign melanocytic nevi. Furthermore, our results are compared to one of the most well-known texture feature extraction methods (co-occurrence matrices).

The rest of the paper is organized as follows: Section 2 introduces the proposed bidimensional colored fuzzy entropy measures; Section 3 presents the validation images used; Section 4 reports the experimental results and their analysis; finally, Section 5 draws the conclusion of this paper.

## 2. Colored Bidimensional Fuzzy Entropy

We recently developed bidimensional fuzzy entropy, $FuzEn_{2D}$, and its multi-scale extension $MSF_{2D}$ [17,18,38]. These entropy measures revealed interesting results for some dermoscopic images but were limited to gray scale images. Based on $FuzEn_{2D}$, we propose herein approaches to deal with colored images: the single-channel bidimensional fuzzy entropy, $FuzEnC_{2D}$ [28] which considers the characteristics of each channel independently, and the multi-channel bidimensional fuzzy entropy measures, $FuzEnV_{2D}$ and $FuzEnM_{2D}$, which take into consideration the inter-channel characteristics. In this paper, we limit our study to three color channels. However, extension to a higher number of channels would be straightforward. For a colored image **U** of *W* width, *H* height, and *K* channels (W×H×K pixels), the following initial parameters are first set: tolerance level *r*, fuzzy power *n*, and window size *m* (see below). The algorithms to compute $FuzEnC_{2D}$, $FuzEnV_{2D}$, and $FuzEnM_{2D}$ are presented below.

### 2.1. $FuzEnC_{2D}$ Single-Channel Approach

The colored image **U** is separated into its corresponding color channels K1, K2, and K3, as **U**${}_{K1}$, **U**${}_{K2}$, and **U**${}_{K3}$, respectively. For each channel composed of $u_{K}\left(i,j\right)$ elements, **X${}_{i,j,K}^{m}$** is designated as the *m*-length square window:

$$\left[\begin{array}{cccc} u_{K}\left(i,j\right) & \cdots & u_{K}\left(i,j+m-1\right)\\ u_{K}\left(i+1,j\right) & \cdots & u_{K}\left(i+1,j+m-1\right)\\ \cdots & \cdots & \cdots & \\ u_{K}\left(i+m-1,j\right) & \cdots & u_{K}\left(i+m-1,j+m-1\right) \end{array}\right],$$

with *K* = K1, K2, or K3 and the indices are defined as such: 1≤i≤H−m and 1≤j≤W−m. The m+1 square window, **X${}_{i,j,K}^{m+1}$**, is defined in the same way. In each of **U**${}_{K1}$, **U**${}_{K2}$, and **U**${}_{K3}$, the total number of defined square windows for both *m* and m+1 sizes is $N_{m}=\left(W-m\right)\left(H-m\right)$.

Based on the original fuzzy entropy definition, $FuzEn_{1D}$[39], a distance function $d_{ij,ab,K}^{m}$ between **X${}_{i,j,K}^{m}$** and its neighboring windows **X${}_{a,b,K}^{m}$** is defined as the maximum absolute difference in their corresponding scalar components. We compose $d_{ij,ab,K}^{m}$ as follows:

(1) $$\begin{array}{c} d_{ij,ab,K}^{m}=d\left[X_{i,j,K}^{m},X_{a,b,K}^{m}\right]\\ \;\;=\max_{s,t\in (0,m-1)}\left(\right|u_{K}\left(i+s,j+t\right)-u_{K}\left(a+s,b+t\right)\left|\right), \end{array}$$

with *a* ranging from 1 to H−m and *b* ranging from 1 to W−m. The similarity degree $D_{ij,ab,K}^{m}$ of **X${}_{i,j,K}^{m}$** with its neighboring patterns **X${}_{a,b,K}^{m}$** is defined by a continuous fuzzy function $\mu (d_{ij,ab,K}^{m},n,r)$:

(2) $$D_{ij,ab,K}^{m}\left(n,r\right)=\mu \left(d_{ij,ab,K}^{m},n,r\right)=\exp\left(-{\left(d_{ij,ab,K}^{m}\right)}^{n}/r\right).$$

Afterwards, the similarity degree of each **X${}_{i,j,K}^{m}$** is averaged to obtain $\Phi_{i,j,K}^{m}\left(n,r\right)$ and then construct:

(3) $$\Phi_{K}^{m}\left(n,r\right)=\frac{1}{N_{m}}\sum_{i=1,j=1}^{i=H-m,j=W-m}\Phi_{i,j,K}^{m}\left(n,r\right).$$

It is similar for m+1 patterns to obtain $\Phi_{K}^{m+1}\left(n,r\right)$. Consequently, $FuzEn_{2D}$ of each channel is calculated as:

(4) $$FuzEnC_{K2D}\left(m,n,r,U_{K}\right)=\ln\frac{\Phi_{K}^{m}\left(n,r\right)}{\Phi_{K}^{m+1}\left(n,r\right)}.$$

Finally, $FuzEnC_{2D}$ is defined in each channel as the natural logarithm of the conditional probability that patterns with m×m similar pixels would remain similar for the next (m+1)×(m+1) pixels in each channel:

(5) $$FuzEnC_{2D}\left(m,n,r,U\right)=\left[FuzEnC_{K1,2D},FuzEnC_{K2,2D},FuzEnC_{K3,2D}\right].$$

**Figure 1:**
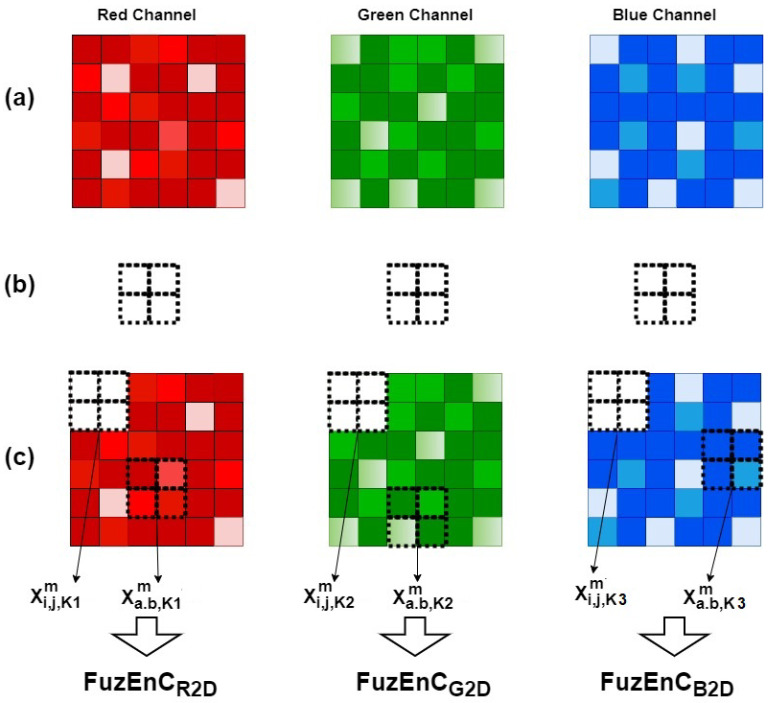
Illustration for $FuzEnC_{2D}$ of an RGB color space image. (**a**) The image **U** is split into its corresponding channels **U**${}_{R}$, **U**${}_{G}$, and **U**${}_{B}$, respectively, from left to right; (**b**) the embedding dimension pattern of size m×m having **m**=[2,2]; (**c**) **X**${}_{i,j,K}^{m}$ and **X**${}_{a,b,K}^{m}$ for K = K1, K2, and K3 being the R, G, and B color channels, respectively.

This single-channel approach treats each channel independently. It has the advantage of allowing us to selectively study certain channels which is of special importance when it comes to images in different color spaces and natures (intensity, color, and texture). In our study, we used n=2. Thus, the similarity degree is expressed by a Gaussian function $\exp(-{\left(d_{ij,ab,K}^{m}\right)}^{2}/r)$. For better illustration, we show in Figure 1 an example for $FuzEnC_{2D}$ of an RGB color space image for an embedding dimension of **m** = [2, 2]; i.e., m×m pixels for each channel. The illustration shows RGB channels as an example, but the same could be applied to different color spaces.

### 2.2. $FuzEnV_{2D}$ Multi-Channel Approach

For an image **U** composed of $u_{i,j,k}$ pixels, **X${}_{i,j,k}^{m}$** is defined as the *m*-length cube. **X${}_{i,j,k}^{m}$** represents the group of pixels in the image **U** of indices from line *i* to i+m−1, column *j* to j+m−1, and the depth of K-channels (k: depth index) as follows:


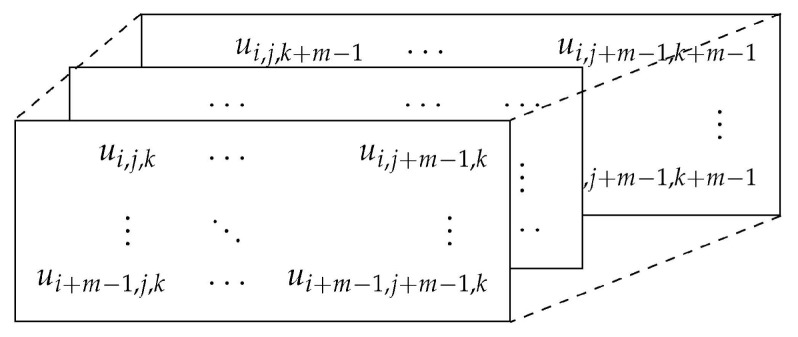


Similarly, **X${}_{i,j,k}^{m+1}$** is defined as the (m+1)-length cube. Let $N_{m}=\left(W-m\right)\left(H-m\right)\left(K-m\right)$ be the total number of cubes that can be generated from **U** for both *m* and m+1 sizes. For **X${}_{i,j,k}^{m}$** and its neighboring cubes **X${}_{a,b,c}^{m}$**, the distance function $d_{ijk,abc}^{m}$ between them is defined as the maximum absolute difference of their corresponding scalar components, knowing that *a*, *b*, and *c* range from 1 to H−m, W−m, and K−m, respectively. Having (a,b,c)≠(i,j,k), the distance function is depicted as follows:

(6) $$\begin{array}{cc} d_{ijk,abc}^{m} & =d\left[X_{i,j,k}^{m},X_{a,b,c}^{m}\right]=\max_{e,f,g\in (0,m-1)}\\ \;(|u(i+e,j+f,k+g)-u(a+e,b+f,c+g)|). \end{array}$$

The similarity degree $D_{ijk,abc}^{m}$ of **X${}_{i,j,k}^{m}$** with its neighboring cubes **X${}_{a,b,c}^{m}$** is defined by a fuzzy function $\mu (d_{ijk,abc}^{m},n,r)$:

(7) $$D_{ijk,abc}^{m}\left(n,r\right)=\mu \left(d_{ijk,abc}^{m},n,r\right)=\exp\left(-{\left(d_{ijk,abc}^{m}\right)}^{n}/r\right).$$

Afterwards, the similarity degree of each cube is averaged to obtain $\Phi_{i,j,k}^{m}\left(n,r\right)$, then construct:

(8) $$\Phi^{m}\left(n,r\right)=\frac{1}{N_{m}}\sum_{i=1,j=1,k=1}^{i=H-m,j=W-m,k=K-m}\Phi_{i,j,k}^{m}\left(n,r\right).$$

This is similar for m+1 cubes to obtain $\Phi^{m+1}\left(n,r\right)$. Finally, multi-channel bidimensional fuzzy entropy of the colored image U is defined as the natural logarithm of the conditional probability that cubes similar in their m×m×m pixels would remain similar for the next (m+1)×(m+1)×(m+1) pixels:

(9) $$FuzEnV_{2D}\left(m,n,r,U\right)=\ln\frac{\Phi^{m}\left(n,r\right)}{\Phi^{m+1}\left(n,r\right)}.$$

**Figure 2:**
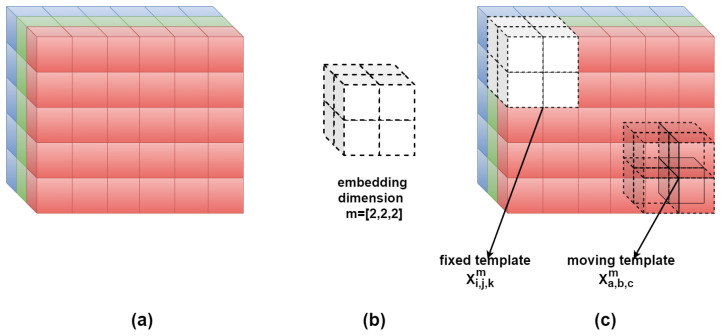
Illustration for $FuzEnV_{2D}$ of an RGB color space image having **m** = [ 2,2,2]. (**a**) A portion of the colored image U with its R, G, and B channels; (**b**) the scanning pattern or embedding dimension with **m**=[2,2,2] that is a 2×2×2 cube; (**c**) **X${}_{i,j,k}^{m}$** and **X${}_{a,b,c}^{m}$**, the fixed and moving templates defined above.

The multi-channel approach has the advantage of extracting inter-channel features. However, we limit our study herein to 3-channel colored images. Thus, the embedding dimension *m* values could be 1 or 2 to avoid exceeding the maximum possible 3×3×3 pixels cubes for the m+1 calculations. This means that for K channels the *m*-value can only be defined between 1 and K-1. Herein, *n* is taken to be 2 and *r* within the range suggested in previous studies. For better illustration, we show in Figure 2 an example for $FuzEnV_{2D}$ of an RGB color space image for an embedding dimension of **m** = [2, 2, 2].

### 2.3. $FuzEnM_{2D}$ Modified Multi-Channel Approach

Since the $FuzEnV_{2D}$ embedding dimension size is limited to *m* = 1 and *m* = 2 for this trichromatic study (K = 3), we introduce herein a modified colored multi-channel approach that can take up to any *m* value. This method is similar to $FuzEnV_{2D}$ except for the fact that the embedding dimension is a cuboid of m×m×K voxels for $FuzEnM_{2D}$. Therefore, the third dimension of the template is not limited by the number of color channels in the study.

For image **U** with K = 3 color channels, composed of $u_{i,j,k}$ voxels, **X${}_{i,j,k}^{m}$** is defined as the m×m×3 cuboid. **X${}_{i,j,k}^{m}$** represents the group of voxels in the image **U** of indices from line *i* to i+m−1, column *j* to j+m−1, and the depth of K-channels (k: depth index). Similarly, **X${}_{i,j,k}^{m+1}$** is defined as the (m+1)×(m+1)×3 cuboid. Let $N_{m}=\left(W-m\right)\left(H-m\right)$ be the total number of cuboids that can be generated from **U** for both *m* and m+1 sizes. Sizes *m* and m+1 stand for [*m*, *m*, 3] and [m+1, m+1, 3] that are made up of m×m×3 and (m+1)×(m+1)×3 voxels, respectively.

For **X${}_{i,j,k}^{m}$** and its neighboring cuboids **X${}_{a,b,c}^{m}$**, the distance function $d_{ijk,abc}^{m}$ between them is defined as the maximum absolute difference of their corresponding scalar components, knowing that *a* and *b* range from 1 to H−m and W−m, respectively, whereas *c* is 1. Having (a,b,c)≠(i,j,k), the distance function is depicted as follows:

(10) $$\begin{array}{c} d_{ijk,abc}^{m}=d\left[X_{i,j,k}^{m},X_{a,b,c}^{m}\right]=\max_{\begin{array}{c} e \end{array},f\in \left(0,m-1\right)g\in \left(0,2\right)}\\ \;(|u(i+e,j+f,k+g)-u(a+e,b+f,c+g)|). \end{array}$$

The similarity degree $D_{ijk,abc}^{m}$ of **X${}_{i,j,k}^{m}$** with its neighboring cuboids **X${}_{a,b,c}^{m}$** is defined by a fuzzy function $\mu (d_{ijk,abc}^{m},n,r)$:

(11) $$D_{ijk,abc}^{m}\left(n,r\right)=\mu \left(d_{ijk,abc}^{m},n,r\right)=\exp\left(-{\left(d_{ijk,abc}^{m}\right)}^{n}/r\right).$$

Afterwards, the similarity degree of each cuboid is averaged to obtain $\Phi_{i,j,k}^{m}\left(n,r\right)$, then construct:

(12) $$\Phi^{m}\left(n,r\right)=\frac{1}{N_{m}}\sum_{i=1,j=1,k=1}^{i=H-m,j=W-m,k=K}\Phi_{i,j,k}^{m}\left(n,r\right).$$

This is similar for (m+1)×(m+1)×3 cuboids to obtain $\Phi^{m+1}\left(n,r\right)$. Finally, multi-channel bidimensional fuzzy entropy of the colored image U is defined as the natural logarithm of the conditional probability that cuboids similar in their m×m×3 voxels would remain similar in their (m+1)×(m+1)×3 voxels:

(13) $$FuzEnM_{2D}\left(m,n,r,U\right)=\ln\frac{\Phi^{m}\left(n,r\right)}{\Phi^{m+1}\left(n,r\right)}.$$

**Figure 3:**
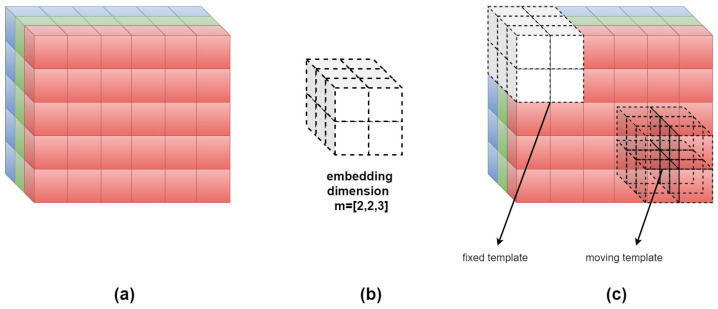
Illustration for $FuzEnM_{2D}$ of RGB color space image having **m**=[2,2,3]. (**a**) A portion of the colored image U with its R, G, and B channels; (**b**) the scanning pattern or embedding dimension with **m**=[2,2,3] that is a 2×2×3 cuboid; (**c**) the fixed and moving templates defined above.

$FuzEnM_{2D}$ has the advantage of extracting inter-channel features and always considering all the color channels of texture images. However, as mentioned previously, we consider our study herein for 3-channel colored images which could be further adapted to a higher number as well. Herein, *n* is taken to be 2 and *r* within the range suggested in previous studies. For better illustration, we show in Figure 3 an example for $FuzEnM_{2D}$ of an RGB color space image for an embedding dimension of **m** = [2, 2, 3]; i.e., moving *m*-sized cuboid is 2×2×3.

### 2.4. Comparing Algorithms

The proposed entropy measures are based on the fuzzy entropy definition [17,39,40] that calculates the similarity degree between the corresponding patterns using a continuous fuzzy function. The latter ensures calculating a participation degree for all the compared patterns and quantifies the irregularity of the analyzed data. This information theory concept has been proven to be reliable for 1D, 2D, and 3D data [17,18,38,39,40]. However, only gray scale data have been investigated to date. Therefore, the idea to analyze colored texture images using the fuzzy entropy concept from a single channel and a multi-channel perspective is interesting.

The major differences between the three proposed algorithms are in the way the similarity degrees are calculated. For the single-channel approach, $FuzEnC_{2D}$, the image is analyzed channel by channel and the result is three entropy values that represent the three channels, respectively, please refer to Figure 1. This is a particular advantage when it comes to analyzing and comparing specific channels in different color spaces. On the other hand, the multi-channel approaches, $FuzEnV_{2D}$ and $FuzEnM_{2D}$, deal with all the channels at the same time; i.e., the inter-channel information is taken into account (unlike handling each color channel separately). $FuzEnV_{2D}$ transforms the 2D similarity degree scanning window into a 3D cubic pattern that studies similarity among the m×m×m and the m+1×m+1×m+1 patterns within a colored image. $FuzEnV_{2D}$ showed good results but for the application in trichromatic color spaces the embedding dimension size was limited to *m* = 1 or 2, please see Figure 2. Therefore, in order to investigate similarity degrees with larger embedding dimension sizes, we present the modified multi-channel approach $FuzEnM_{2D}$, please refer back to Figure 3. $FuzEnC_{2D}$, $FuzEnV_{2D}$, and $FuzEnM_{2D}$ provide colored texture analysis from single-channel and multi-channel perspectives. The choice of the algorithm depends on the intended application. Moreover, the analysis could be extended to multi-spectral images and even other color spaces than the ones discussed in this paper.

## 3. Validation Tests and Medical Database

In order to validate the proposed colored bidimensional entropy measures, we studied their sensitivity to different parameter values. The algorithms were also tested using images with different degrees of randomness and the colored Brodatz dataset [41]. The images were normalized by subtracting their mean and dividing by their standard deviation and all the tests were performed using Matlab. In the following, we describe the elements used for the validation tests and the medical dataset.

### 3.1. MIX${}_{2D}$(p) Processes

MIX${}_{2D}$(*p*) [12] is a family of images of stochastic processes that are moderated by the probability of irregularity, *p*, varying from 0 (totally regular periodic image) to 1 (totally irregular image). We used MIX${}_{2D}$(*p*) for the single-channel approach, and MIX${}_{3D}$(*p*), a volumetric extension for MIX${}_{2D}$(*p*) proposed by [40], for our multi-channel approach.

### 3.2. Colored Brodatz Images

**Figure 4:**
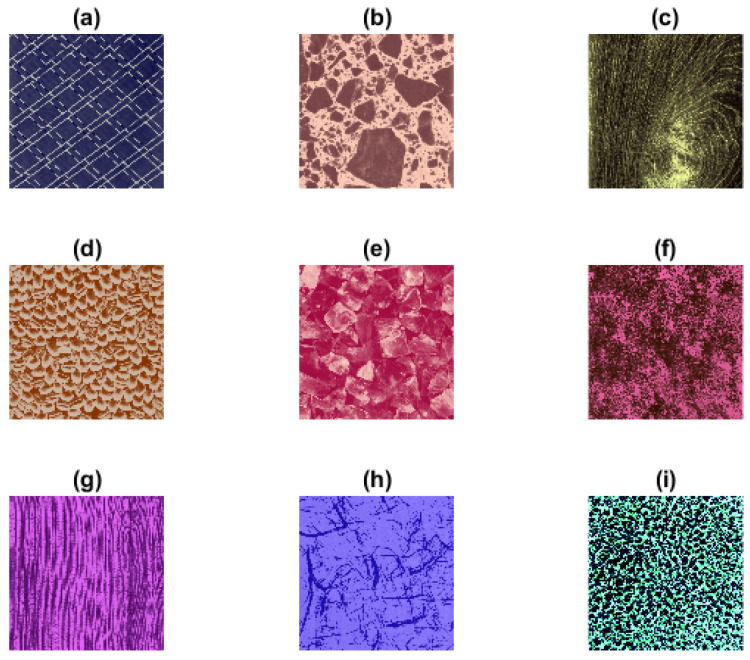
Colored Brodatz texture (CBT) images of different colored irregularity degrees [41,42]. (**a**–**i**) CBT images that are used for the validation test (Section 4.3) to compare the entropy values of each colored texture to its corresponding sub-images in three color spaces (RGB, HSV, and YUV); (**f**) is used again for studying the sensitivity of the proposed measures to different initial parameters (Section 4.1).

For texture validation tests, we used the colored Brodatz texture (CBT) [41,42] images, see Figure 4. CBT presents colored textures with different degrees of visible irregularity. We can notice that, for example, the CBT images (a), (b) and (e) show more regular and periodic repetitive patterns than (c), (f) and (i).

### 3.3. Color Spaces

Besides using the most common trichromatic color space, red, green, blue (RGB), we extend our study by transforming the images to use two other color spaces: hue, saturation, value (HSV; hue and saturation: chrominance, value: intensity) and YUV (Y: luminance, U and V: chrominance) to investigate the effect of color space transformations on $FuzEnC_{2D}$, $FuzEnM_{2D}$, and $FuzEnM_{2D}$ outcomes. In RGB color space, the intensity and color are combined to give us the final display, whereas for HSV and YUV color spaces, intensity and color are separated.

### 3.4. Co-Occurrence Matrices

For the application on medical images, we study the effect of different color spaces and compare our results to those obtained with gray level co-occurrence matrices [43], which probably remains the most used texture analysis technique. We employed the co-occurrence matrices of each channel (integrative way) for comparing the results to our single-channel approach, and its extended 3D co-occurrence matrices [44] for comparing the results to our multi-channel approach. We thus adopted the following procedure:

- The 2D co-occurrence matrices were created considering 4 orientations (0${}^{\circ }$, 45${}^{\circ }$, 90${}^{\circ }$, and 135${}^{\circ }$), 4 inter-pixel distances (1, 2, 4, and 8), and 8 gray levels ($N_{g}$ = 8) to be compared with $FuzEnC_{2D}$.
- The 3D co-occurrence matrices were created considering 13 orientations [44], 4 inter-pixel distances (1, 2, 4, and 8), and 8 gray levels to be compared with $FuzEnV_{2D}$ and $FuzEnM_{2D}$.

Then, we calculated the Haralick features for each co-occurrence matrix (for each orientation and distance). Finally, the average of features for all matrices was calculated to be compared with $FuzEnC_{2D}$, $FuzEnV_{2D}$, and $FuzEnM_{2D}$ values. Among the 14 features originally proposed [43], only six are commonly employed by researchers due to their correlation with the other eight, see Table 1.

### 3.5. Medical Images

**Figure 5:**
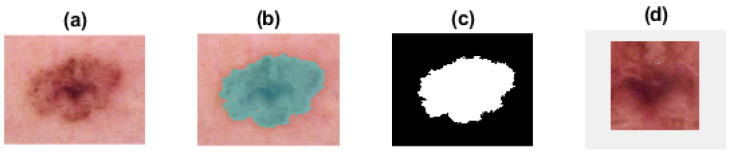
Dermoscopic images segmentation for choosing the region of interest (ROI). (**a**) an example of the dermoscopic image for a pigmented skin lesion; (**b**,**c**) the contouring and segmentation of the lesion; (**d**) the ROI as the central 128×128×3 pixels.

For our medical application we used the HAM10000, “Human Against Machine with 10,000 training images” [45,46]. The dataset is composed of dermoscopic images for pigmented lesions, see an example in Figure 5a. The dataset contains dermoscopic images of melanocytic nevi, melanoma, dermatofibroma, actinic keratoses, basal cell carcinoma, and benign keratosis [45].

As suggested by medical doctors, the most significant comparison is that between melanoma and melanocytic nevi. The target of the medical application in our study is to try to differentiate the deadliest type of skin cancer, melanoma, from the benign melanocytic nevi. These two widespread types of pigmented skin lesions are often mistaken in diagnosis and detection, especially in their early stages. Moreover, early diagnosis and excision could vastly increase the patients’ survival rate [29,30,31]. Thus, we selected from the proposed dataset forty melanoma images and forty melanocytic nevi images to be processed and compared.

## 4. Results and Discussion

In this section, we present the results of the validation tests. We start by testing the algorithms’ sensitivity to initial parameter choice, then we explore the algorithms’ ability to identify increasing irregularity degrees in colored textures. After that, we analyze colored Brodatz texture images in 3 different color spaces (RGB, YUV, and HSV). Finally, we show the results using $FuzEnC_{2D}$, $FuzEnV_{2D}$, and $FuzEnM_{2D}$ for melanoma and melanocytic nevi dermoscopic images and compare them to those obtained using single-channel and multi-channel co-occurrence matrices.

### 4.1. Sensitivity to Initial Parameters

To study the sensitivity of our proposed measures, with different embedding dimensions *m* and tolerance levels *r*, we evaluated 100 × 100 pixels of a colored Brodatz image (Figure 4f) using different parameter choices.

**Figure 6:**
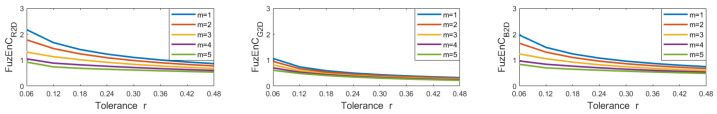
$FuzEnC_{2D}$ results for the red, green, and blue channels (left to right) of the colored Brodatz image, Figure 4f, with varying *r* and *m*.

**Figure 7:**
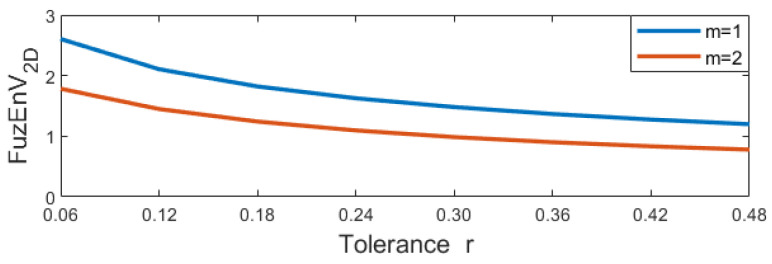
$FuzEnV_{2D}$ results with varying *r* and *m* of the colored Brodatz image, Figure 4f.

**Figure 8:**
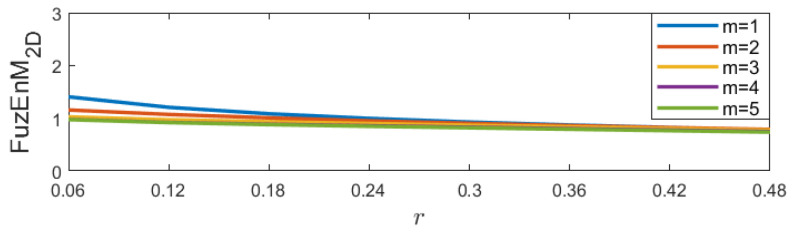
$FuzEnM_{2D}$ results with varying *r* and *m* of the colored Brodatz image, Figure 4f.

- For $FuzEnC_{2D}$, the embedding dimension *m* was taken as 1, 2, 3, 4, and 5, and the tolerance level *r* from 0.06 up to 0.48 (step 0.06). The results are displayed in Figure 6.
- For $FuzEnV_{2D}$, the embedding dimension *m* was taken as 1 and 2, since the maximum possible cube volume for (m+1)-length cubes is 3×3×3 pixels (given the 3 color channels). The results are displayed in Figure 7.
- For $FuzEnM_{2D}$, the embedding dimension *m* was taken as 1, 2, 3, 4, and 5, and the tolerance level *r* from 0.06 up to 0.48 (step 0.06). The results are displayed in Figure 8.

We observe that $FuzEnC_{2D}$, $FuzEnV_{2D}$, and $FuzEnM_{2D}$ remain defined for different chosen initial parameters. Additionally, the algorithms show low variability upon changes in *r* and *m*. This illustrates their low sensitivity to *r* and *m*, allowing a certain degree of freedom in our choice of initial parameters without restrictions.

### 4.2. Detecting Colored Image Irregularity

We generated 256×256 pixel MIX${}_{2D}$(*p*) in three channels and 256×256×3 pixel MIX${}_{3D}$(*p*) images and analyzed them by single-channel ($FuzEnC_{2D}$) and multi-channel approaches ($FuzEnV_{2D}$ and $FuzEnM_{2D}$), respectively.

**Figure 9:**
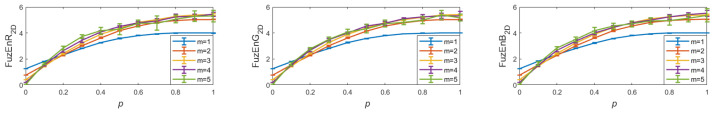
$FuzEnC_{2D}$ mean and standard deviation for MIX${}_{2D}$(*p*) images with 10 repetitions.

**Figure 10:**
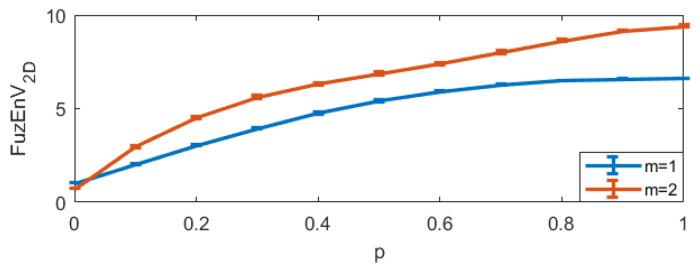
$FuzEnV_{2D}$ mean and standard deviation for MIX${}_{3D}$(*p*) images with 10 repetitions.

**Figure 11:**
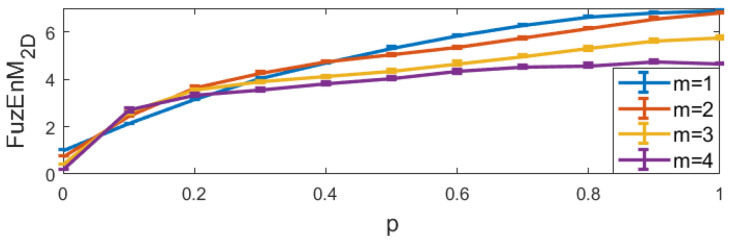
$FuzEnM_{2D}$ mean and standard deviation for MIX${}_{3D}$(*p*) images.

- $FuzEnC_{2D}$: we set r=0.15, m=1,2,3,4,5, and p=0 to 1 with a step of 0.1, and repeated the calculation for 10 images each. The results are depicted in Figure 9.
- $FuzEnV_{2D}$: we set r=0.15, m=1 and2 (as the maximum possible cube volume for m+1 could only be 3×3×3 pixels), p=0 to 1 with a step of 0.1, and repeated the calculation for 10 images each. The results are depicted in Figure 10.
- $FuzEnM_{2D}$: we set r=0.15, *m*=1,2,3, and4, and p=0 to 1 with a step of 0.1, and repeated the calculation for 10 images each. The results are depicted in Figure 11.

The results show that both the single- and multi-channel approaches lead to increasing entropy values with increasing irregularity degree, *p*. This illustrates their ability to properly quantify increasing irregularity degrees and their consistency upon repetition.

### 4.3. Studying Texture Images

**Figure 12:**
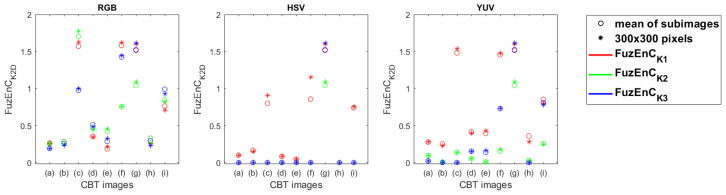
$FuzEnC_{2D}$ results for the 144 sub-images and 300 × 300 pixels of the CBT in the three color spaces: RGB, HSV, and YUV, with K1, K2, and K3 being the first, second, and third channel, respectively. The mean of the 144 sub-images is displayed as a “∘” sign and the value for the 300 × 300 pixels is displayed as “*”.

**Figure 13:**
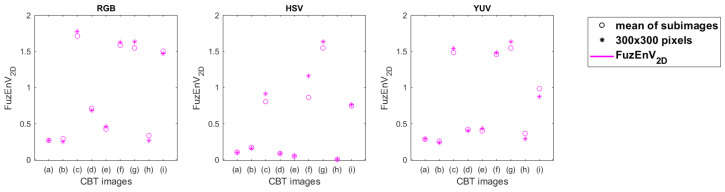
$FuzEnV_{2D}$ results for the 144 sub-images and 300 × 300 pixels of the CBT in the three color spaces: RGB, HSV, and YUV. The mean of the 144 sub-images is displayed as a “∘” sign and the value for the 300 × 300 pixels is displayed as “*”.

Nine CBT [41,42] images of 640 × 640 pixels, see Figure 4, were split into 144 sub-images of size 50 × 50 pixels. $FuzEnC_{2D}$, $FuzEnV_{2D}$, and $FuzEnM_{2D}$ were calculated for these sub-images and for a 300 × 300 pixel corner region from each corresponding original CBT image. The parameters *r* and *m* were set to 0.15 and 2, respectively. The results with $FuzEnC_{2D}$ and $FuzEnV_{2D}$ are depicted in Figure 12 and Figure 13. Similar results to those of $FuzEnV_{2D}$ are found with $FuzEnM_{2D}$. We observe that, especially for the RGB color space, most of the $FuzEnC_{2D}$, $FuzEnV_{2D}$, and $FuzEnM_{2D}$ averages of the sub-images overlap with or are very similar to the value of their corresponding image’s 300 × 300 pixel region. Moreover, we notice their differentiation ability between different CBT images. In the HSV and YUV color spaces, the multi-channel approaches outperform $FuzEnC_{2D}$ (Figure 12) in differentiating the CBT images. We can also observe that for the RGB color space, the CBT images that are perceived visually to be of higher color and pattern irregularity, Figure 4c,f,g, obtained higher entropy values than the others, whereas those that appear to be of periodic well-defined repetitive patterns, Figure 4a,b,e, resulted in lower entropy values for the three measures $FuzEnC_{2D}$, $FuzEnV_{2D}$, and $FuzEnM_{2D}$. This is in accordance with the literature of entropy measures and information theory concept applied to gray level texture images [12,14,15,16,17,18,38].

### 4.4. Medical Image Analysis

We calculated $FuzEnC_{2D}$, $FuzEnV_{2D}$, and $FuzEnM_{2D}$ for 40 melanoma images and 40 melanocytic nevi images from the HAM10000 dataset [45] in the color spaces RGB, HSV, and YUV. In order to determine the region of interest (ROI) of melanoma and melanocytic nevi images, the lesions were segmented as shown in Figure 5. Then, the central region of 128×128×3 pixels was selected, see Figure 5d. By adopting this procedure, we ensured that the same number of pixels were processed (equally sized images) and that no region outside the lesion was included. The parameters *r* and *m* were set to 0.15 and 2, respectively. The images were normalized by subtracting their mean and dividing by their standard deviation.

To validate the statistical significance of $FuzEnC_{2D}$, $FuzEnV_{2D}$, and $FuzEnM_{2D}$ in differentiating melanoma from melanocytic nevi images, we used the Mann–Whitney U test. The resulting *p*-values are presented in Table 2. $FuzEnC_{2D}$ shows statistical significance (for *p* < 0.05) in differentiating melanoma and melanocytic nevi for all the channels except V (of HSV color space). In addition, using $FuzEnV_{2D}$ and $FuzEnV_{2D}$, melanoma and melanocytic nevi images are identified as statistically different for the three color spaces. Moreover, we calculated the Cohen’s *d* [47,48] to further validate our obtained statistical results, see Table 3. Most *d* values reflect “large”, “very Large”, and “huge” effect sizes, which validates the differentiation ability of our proposed measures.

**Figure 14:**
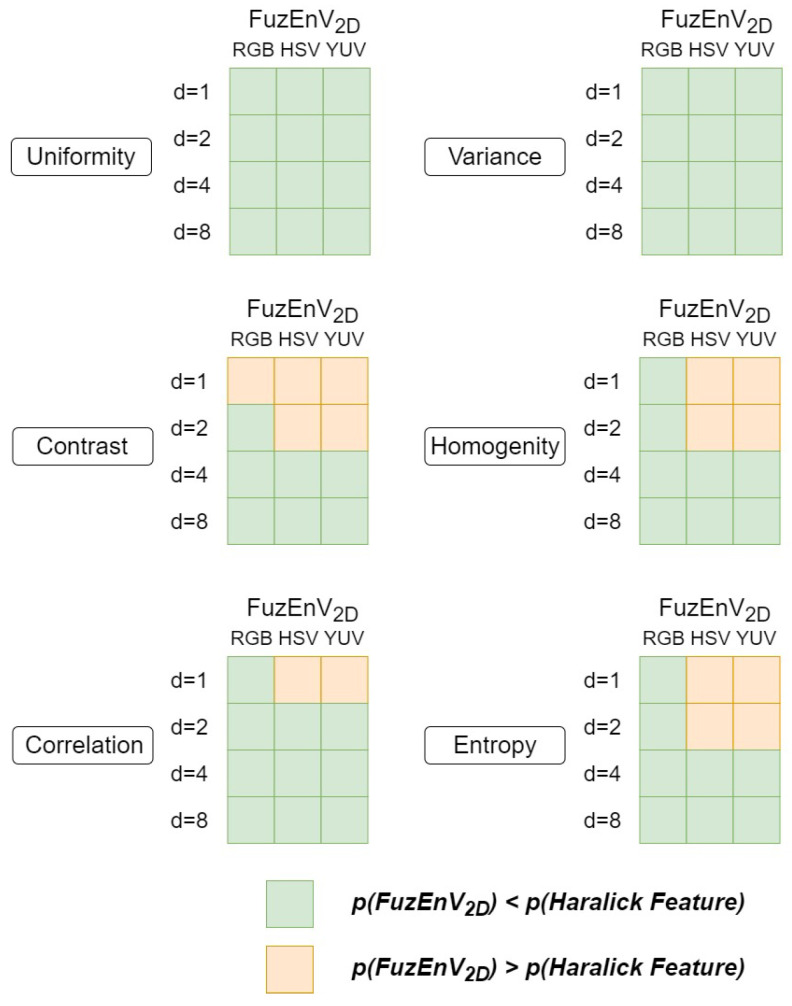
$FuzEnV_{2D}$ and Haralick feature *p*-values of 40 melanoma and 40 melanocytic nevi dermoscopic images in the 3 color spaces: RGB, HSV, and YUV. *d* represents the inter-pixel distances for the co-occurrence matrices.

**Figure 15:**
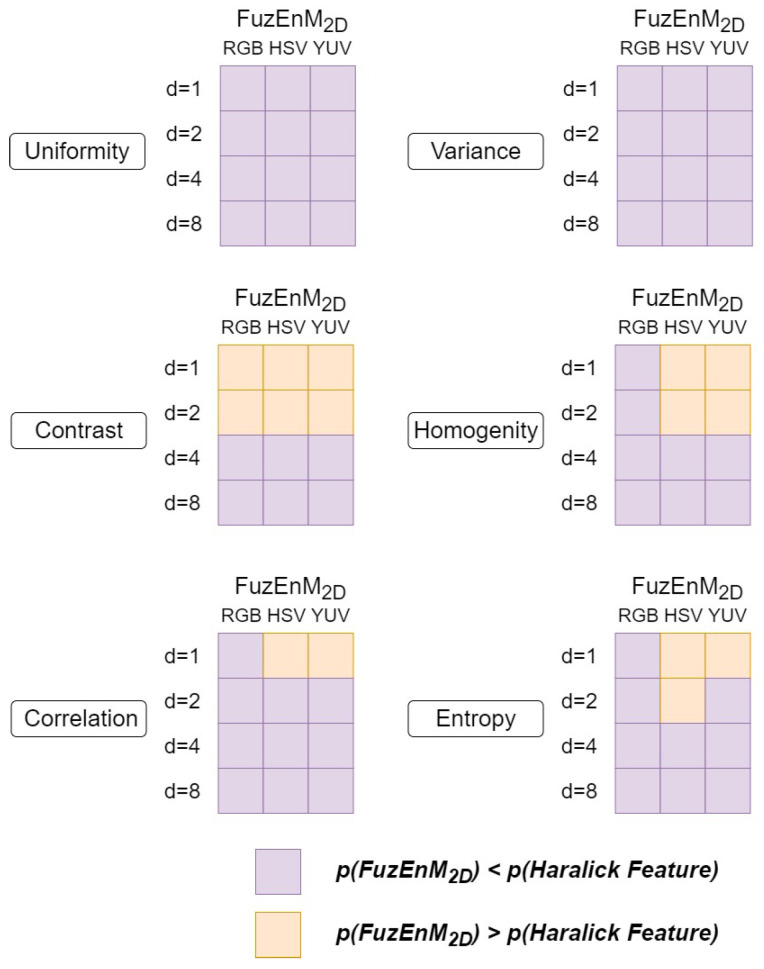
$FuzEnM_{2D}$ and Haralick feature *p*-values of 40 melanoma and 40 melanocytic nevi dermoscopic images in the 3 color spaces: RGB, HSV, and YUV. *d* represents the inter-pixel distances for the co-occurrence matrices.

Additionally, we compared $FuzEnC_{2D}$ results with Haralick features from 2D co-occurrence matrices. The results show that $FuzEnC_{2D}$ results in lower *p*-values than Haralick features for the G, H, Y, and U channels and none of the methods result in statistical significance for the S channel. Additionally, we compared $FuzEnV_{2D}$ and $FuzEnM_{2D}$ results with Haralick features from 3D co-occurrence matrices. The summaries of results for $FuzEnV_{2D}$ and $FuzEnM_{2D}$ are shown in Figure 14 and Figure 15, respectively. $FuzEnV_{2D}$ and $FuzEnM_{2D}$ surpassed Haralick features as *p*-values obtained for the results of both entropy measures are mostly lower than those of Haralick features. Moreover, using Haralick features, some results do not show statistical significance (*p* > 0.05), whereas all the three proposed colored entropy measures illustrate evident statistical significance in differentiating melanoma from melanocytic nevi, except in $FuzEnC_{2D}$ results for S and V color channels.

**Figure 16:**
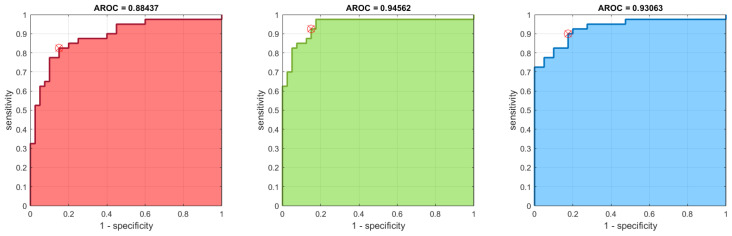
ROC curves for $FuzEnC_{2D}$ results of the 40 melanoma and 40 melanocytic nevi images in the RGB color space. The curves are for $FuzEnC_{R2D}$, $FuzEnC_{G2D}$, and $FuzEnC_{B2D}$ from left to right.

**Figure 17:**
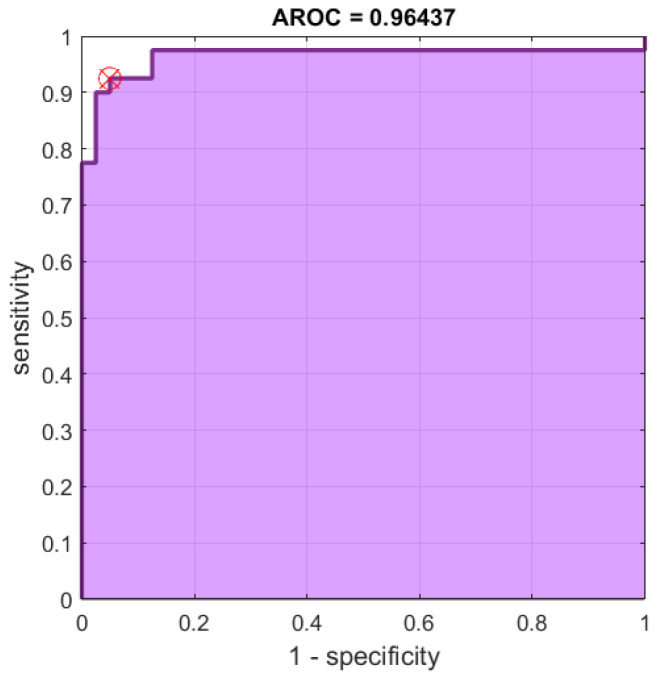
ROC curves for $FuzEnV_{2D}$ results of the 40 melanoma and 40 melanocytic nevi images in the RGB color space.

**Figure 18:**
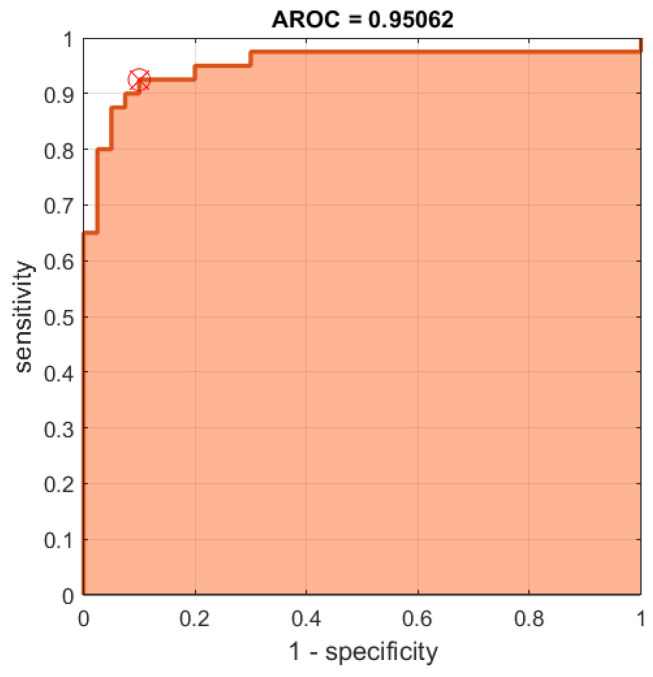
ROC curves for $FuzEnM_{2D}$ results of the 40 melanoma and 40 melanocytic nevi images in the RGB color space.

In addition to the *p*-value test, the receiver operating characteristic (ROC) and area under the ROC curve (AUC) of the results can be used as a criterion to measure the discrimination ability of our proposed measures. Since the best results (lowest *p*-values) were obtained for the RGB color space, we further establish the ROC curves for its $FuzEnC_{2D}$, $FuzEnV_{2D}$, and $FuzEnM_{2D}$ results, see Figure 16, Figure 17 and Figure 18, respectively. Moreover, the AUC, sensitivity, specificity, accuracy, and precision are shown for the RGB, HSV, and YUV color spaces in Table 4, Table 5 and Table 6, respectively. The results show that $FuzEnC_{2D}$ has high accuracy and AUC values for R, G, B, H, Y, U, and V channels. In addition, the multi-channel approaches ($FuzEnV_{2D}$ and $FuzEnM_{2D}$) illustrate high accuracy and AUC values for the three color spaces. For the three proposed entropy measures, the best accuracy and AUC values were obtained for the RGB color space.

Finally, we can say that the three entropy measures were able to differentiate both pigmented skin lesions. This was validated statistically by *p*-values, especially in the RGB color space. In the latter, $FuzEnC_{2D}$ achieved accuracies of 83.7%, 88.7%, 86.2% and AUC of 88.4%, 94.5%, 93%. On the other hand, $FuzEnV_{2D}$, resulted in an accuracy of 93.7% and AUC of 96.4%. In addition, $FuzEnM_{2D}$ showed an accuracy of 91.2% and AUC of 95.0%.

## 5. Conclusions

In this paper, we presented a new concept and the first entropy method to investigate the single- and multi-channel features of colored images. To the best of our knowledge, this study is the only one that suggests entropy measures for analyzing colored images in their single- and multi-channel approaches. It was essential to perform some validation tests before employing those measures for analyzing colored medical images. The study was carried out as follows:

- Studying the sensitivity of the proposed measures to different initial parameters (tolerance level *r* and window size *m*).
- Identifying different irregularity degrees in colored images.
- Studying colored texture images in three color spaces.
- Analyzing medical images in three color spaces.

The three entropy measures, $FuzEnC_{2D}$, $FuzEnV_{2D}$, and $FuzEnM_{2D}$, showed a reliable behavior with different initial parameters and an ability to gradually quantify irregularity degrees of colored textures and consistency upon repetition. When considering different color spaces, RGB, HSV, and YUV, these entropy measures showed promising results for the colored texture images.

Regarding the dermoscopic melanoma and melanocytic nevi images, single- and multi-channel entropy measures were able to differentiate both pigmented skin lesions. This was validated statistically by *p*-values, especially in the RGB color space. In the latter, $FuzEnC_{2D}$ achieved accuracies of 83.7%, 88.7%, 86.2% and AUC of 88.4%, 94.5%, 93%. On the other hand, $FuzEnV_{2D}$, reached an accuracy of 93.7% and AUC of 96.4%. In addition, $FuzEnM_{2D}$ showed an accuracy of 91.2% and AUC of 95.0%. Moreover, $FuzEnV_{2D}$ and $FuzEnM_{2D}$ outperformed both $FuzEnC_{2D}$ and the classical descriptors, Haralick features, in differentiating the two similar malignant melanoma and benign melanocytic nevi dermoscopic images. These preliminary results could be the groundwork for developing an objective computer-based tool for helping medical doctors in diagnosing melanoma that is often mistaken for a benign melanocytic nevi or is properly diagnosed only in its late stages. We limited our investigation to three-channel colored images and, consequently, future work could be directed towards multi-spectral color images and towards more adapted applications for each color space and extending our study to a larger dataset. 

## Figures and Tables

**Table 1:** Definition of the computed Haralick features [43].

Haralick Feature	Annotation
Uniformity (Energy)	$\sum_{i}\sum_{j}P^{2}\left(i,j\right)$
Contrast	$\sum_{n=0}^{N_{g}-1}n^{2}\left(\sum_{i=1}^{N_{g}}\sum_{j=1}^{N_{g}}P\left(i,j\right)\right),\left|i-j\right|=n$
Correlation	$\sum_{i}\sum_{j}\left(ij\right)P\left(i,j\right)-\mu_{x}\mu_{y}/\sigma_{x}\sigma_{y}$
Variance	$\sum_{i}\sum_{j}{\left(i-\mu \right)}^{2}P\left(i,j\right)$
Homogeneity	$\sum_{i}\sum_{j}P\left(i,j\right)/(1+\left({\left(i-j\right)}^{2}\right)$
Entropy	$-\sum_{i}\sum_{j}P\left(i,j\right)log\;P\left(i,j\right)$

where *P* represents the elements of the co-occurrence matrices and *μ_x_*, *μ_y_*, *σ_x_*, and *σ_y_* are the means and standard deviations of row and column sums, respectively.

**Table 2:** Mann–Whitney U test *p*-values for $FuzEnC_{2D}$, $FuzEnV_{2D}$, and $FuzEnM_{2D}$ of 40 melanoma and 40 melanocytic nevi dermoscopic images in the 3 color spaces: RGB, HSV, and YUV, from top to bottom row, respectively.

${\mathrm{FuzEnC}}_{2D}$			${\mathrm{FuzEnV}}_{2D}$	${\mathrm{FuzEnM}}_{2D}$
$U_{K1}$	$U_{K2}$	$U_{K3}$	U	U
3.3 × $10^{-9}$	7.0 × $10^{-12}$	3.4 × $10^{-11}$	9.0 × $10^{-13}$	4.1 × $10^{-12}$
2.9 × $10^{-5}$	5.7 × $10^{-2}$	1.5 × $10^{-1}$	2.9 × $10^{-5}$	2.9 × $10^{-5}$
9.8 × $10^{-6}$	1.7 × $10^{-3}$	5.8 × $10^{-4}$	4.5 × $10^{-5}$	1.1 × $10^{-5}$

**Table 3:** Cohen’s *d*-values for $FuzEnC_{2D}$, $FuzEnV_{2D}$, and $FuzEnM_{2D}$ of 40 melanoma and 40 melanocytic nevi dermoscopic images in the 3 color spaces: RGB, HSV, and YUV.

	${\mathrm{FuzEnC}}_{2D}$			${\mathrm{FuzEnV}}_{2D}$	${\mathrm{FuzEnM}}_{2D}$
	$U_{K1}$	$U_{K2}$	$U_{K3}$	U	U
RGB	1.50	1.89	1.97	2.71	2.19
HSV	1.14	0.23	0.27	1.14	1.14
YUV	1.10	0.58	0.70	1.00	1.09

**Table 4:** ROC analysis for $FuzEnC_{2D}$, $FuzEnV_{2D}$, and $FuzEnM_{2D}$ results of 40 melanoma and 40 melanocytic nevi RGB images.

	${\mathrm{FuzEnC}}_{2D}$			${\mathrm{FuzEnV}}_{2D}$	${\mathrm{FuzEnM}}_{2D}$
	$U_{R}$	$U_{G}$	$U_{B}$	U	U
AUC	0.884	0.945	0.930	0.964	0.950
Sensitivity	0.825	0.925	0.900	0.925	0.925
Specificity	0.850	0.850	0.825	0.950	0.900
Accuracy	0.837	0.887	0.862	0.937	0.912
Precision	0.846	0.860	0.837	0.948	0.902

**Table 5:** ROC analysis for $FuzEnC_{2D}$, $FuzEnV_{2D}$, and $FuzEnM_{2D}$ results of 40 melanoma and 40 melanocytic nevi HSV images.

	${\mathrm{FuzEnC}}_{2D}$			${\mathrm{FuzEnV}}_{2D}$	${\mathrm{FuzEnM}}_{2D}$
	$U_{H}$	$U_{S}$	$U_{V}$	U	U
AUC	0.771	0.376	0.406	0.771	0.771
Sensitivity	0.650	0.325	0.225	0.650	0.650
Specificity	0.850	0.600	0.850	0.850	0.850
Accuracy	0.750	0.462	0.5375	0.750	0.750
Precision	0.812	0.448	0.600	0.812	0.812

**Table 6:** ROC analysis for $FuzEnC_{2D}$, $FuzEnV_{2D}$, and $FuzEnM_{2D}$ results of 40 melanoma and 40 melanocytic nevi images in YUV.

	${\mathrm{FuzEnC}}_{2D}$			${\mathrm{FuzEnV}}_{2D}$	${\mathrm{FuzEnM}}_{2D}$
	$U_{Y}$	$U_{U}$	$U_{V}$	U	U
AUC	0.787	0.703	0.723	0.765	0.785
Sensitivity	0.725	0.750	0.700	0.750	0.725
Specificity	0.750	0.650	0.700	0.725	0.750
Accuracy	0.737	0.700	0.700	0.737	0.737
Precision	0.743	0.681	0.700	0.731	0.743

## Data Availability

Data sharing not applicable.
